# Basketball Game-Related Statistics that Discriminate among Continental Championships for Under-18 Women

**DOI:** 10.3390/sports6040114

**Published:** 2018-10-10

**Authors:** Haruhiko Madarame

**Affiliations:** Department of Sports and Fitness, Shigakkan University, Nakoyama 55, Yokonemachi, Obu, Aichi 474-8651, Japan; madarame-tky@umin.ac.jp; Tel.: +81-562-46-1291

**Keywords:** match analysis, basketball, women’s sports, regional difference

## Abstract

The purposes of this study were (a) to evaluate differences in basketball game-related statistics among continental championships for under-18 (U18) women, and (b) to identify game-related statistics that discriminate among the continents. The analysis was performed on all matches (*n* = 136) in the four continental championships (Africa, America, Asia, Europe) of 2016. Differences in game-related statistics among the continents were analyzed by an analysis of variance (ANOVA) with effect size statistics. Game-related statistics that discriminate among the continents were assessed by discriminant analysis. The ANOVA yielded significant *F*-values for 13 of 16 variables and large effect size differences for 10 of 16 variables. The discriminant analysis yielded three significant functions. The Asian championship was discriminated from the other continental championships by ball possessions, defensive rebounds, assists, and fouls. The African championship was discriminated from the European championship by ball possessions, successful 3-point field goals, unsuccessful free throws, and turnovers, and from the American championship by ball possessions, unsuccessful 2-point field goals, successful 3-point field goals, successful free throws, and assists. The results of this study suggest that U18 women’s basketball games are played differently in each continent.

## 1. Introduction

Assessing differences in performance profiles among regions of the world is one of the recent topics in the field of basketball performance analysis, and a series of papers have been published in academic journals in recent years [1,2,3,4,5]. Two studies, one on men’s championships [1] and the other on women’s championships [2], compared game-related statistics that discriminate winning teams from losing teams in Asian championships with those in European championships. Ibáñez et al. [3] expanded the scope of study to include other regions of the world, comparing game-related statistics among men’s continental championships and identifying game-related statistics that discriminate among the continents. Following this study, continental championships for women [4] and under-18 (U18) men [5] have also been studied using the same analytical method. From these studies, we can illustrate similarities and differences in performance profiles among regions of the world with age and sex differences taken into account.

Although differences in basketball performance profiles among continental championships have been studied in different categories (men, women, and junior men), continental championships for junior women have yet to be studied. If there are no age and sex interactions in performance profiles, performance profiles of junior women can be inferred from those of men, women, and junior men. However, age and sex interactions in performance profiles have been demonstrated in previous researches [6,7]. Sampaio and colleagues [6] investigated senior and junior world basketball championships for both sexes by using discriminant analysis and reported that significant functions were obtained for age and sex interactions. Therefore, we cannot infer performance profiles among continental championships for junior women from previous studies on men, women, and junior men.

From a practical point of view, the knowledge about regional differences in performance profiles among continental championships for junior women would be useful when players and coaches of junior women’s national teams prepare for matches in international tournaments. In addition, this study would be valuable as part of scientific knowledge in the field of long-term development of female basketball players. Although studies on women’s basketball have been increasing [2,4,8,9,10,11,12,13], studies on junior women are limited [6,7,14]. Therefore, this study aimed (a) to evaluate differences in basketball game-related statistics among continental championships for U18 women, and (b) to identify game-related statistics that discriminate among the continents.

## 2. Materials and Methods

This study analyzed all 136 matches in the four (Africa, America, Asia, Europe) U18 women’s continental championships of 2016 (Table 1). All data were gathered from box scores provided by the official website of the International Basketball Federation (FIBA).

This study did not assess inter-rater reliability of the official box score. However, the official box score has been considered as a reliable data source in basketball researches [15,16]. This is because (i) the recording procedure is stipulated by FIBA statisticians’ manual [17], and (ii) substantial reliability between raters has been demonstrated in previous studies [3,18,19,20]. The following statistics were gathered: 2- and 3-point field goals (both successful and unsuccessful), free throws (both successful and unsuccessful), defensive and offensive rebounds, assists, steals, turnovers, blocks, and fouls committed. The statistics were normalized to 100 game ball possessions [21]. Game ball possessions were calculated as the average value of team ball possessions (TBP) of winning and losing teams [22]. TBP was derived from field goal attempts (FGA), offensive rebounds (ORB), turnovers (TO), and free throw attempts (FTA) using the following formula [22]:

TBP = FGA − ORB + TO + 0.4 × FTA(1)

The statistical software R (version 3.5.0 for Windows, R Foundation for Statistical Computing, Vienna, Austria) [23] was used for the analysis. A level of significance was set at *p* ≤ 0.05. Differences in game-related statistics among the continents were analyzed by one-way ANOVA with Bonferroni-corrected post hoc comparisons. The magnitude of effect sizes for post hoc comparisons was assessed by Cohen’s *d* statistic (*d* = 0.20–0.49, small effect size; *d* = 0.50–0.79, medium effect size; *d* > 0.79, large effect size) [24]. Discriminant analysis was conducted using R code written by Aoki [25,26], and the structural coefficient (SC) was used for identifying game-related statistics that discriminate among the continents (|SC| ≥ 0.30).

## 3. Results

The ANOVA yielded significant *F*-values for points scored, point difference, ball possessions, unsuccessful 2-point field goals, successful 3-point field goals, successful and unsuccessful free throws, defensive and offensive rebounds, assists, steals, turnovers, and fouls (Table 2). Large effect size differences between continents were found in point difference (Africa-Europe, America-Europe, Asia-Europe), ball possessions (Africa-Asia, Africa-Europe, America-Asia, America-Europe, Asia-Europe), unsuccessful 2-point field goals (America-Asia), successful 3-point field goals (Africa-America, Africa-Europe), successful free throws (America-Asia), unsuccessful free throws (Africa-Asia, Africa-Europe), defensive rebounds (Africa-Asia, Asia-Europe), assists (America-Asia, Asia-Europe), turnovers (Africa-Europe), and fouls (Africa-Asia, America-Asia, Asia-Europe).

**Figure 1:**
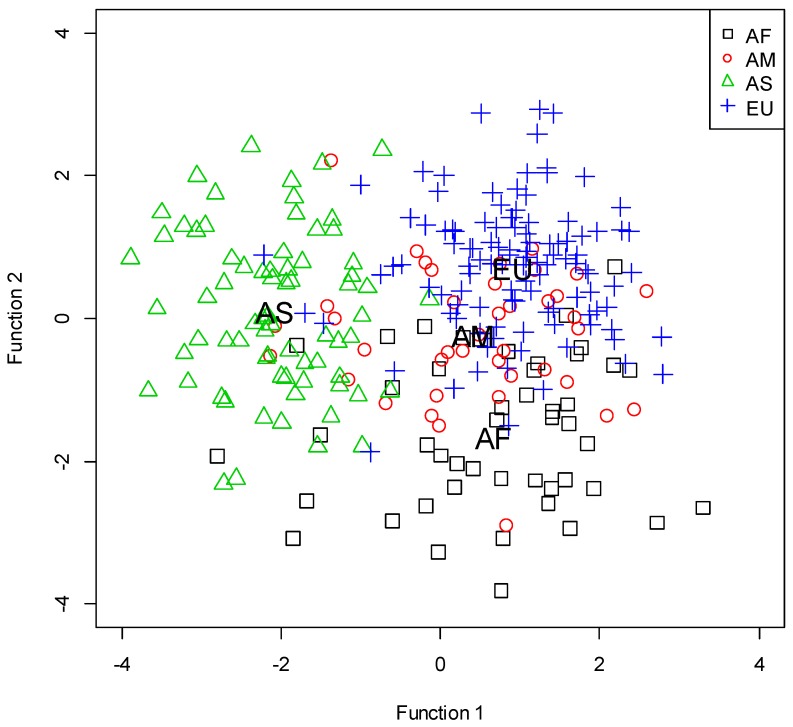
Territorial map of discriminant functions 1 and 2. AF, Africa; AM, America; AS, Asia; EU, Europe. Abbreviations plotted inside the figure represent group centroids.

The results of the classification are presented in Table 3. The overall classification accuracy was 77.9%. The discriminant analysis yielded three discriminant functions (Table 4). The value of Wilks’ Lambda ranges from 0 to 1, and the lower the value, the higher is the discriminating ability. A significant chi-square means that the null hypothesis that the function has no discriminating ability can be rejected. Therefore, the results were interpreted that all the three functions have the ability to discriminate among continents, and the ability is high in the order of the functions (Function 1 > Function 2 > Function 3). Function 1 discriminated between the Asian championship and the other continental championships. The discriminating game-related statistics were ball possessions, defensive rebounds, assists, and fouls. Function 2 discriminated between the African championship and the European championship. The discriminating game-related statistics were ball possessions, successful 3-point field goals, unsuccessful free throws, and turnovers. Function 3 discriminated between the African championship and the American championship. The discriminating game-related statistics were ball possessions, unsuccessful 2-point field goals, successful 3-point field goals, successful free throws, and assists. The territorial map of functions 1 and 2 is presented in Figure 1.

## 4. Discussion

The purposes of this study were (a) to evaluate differences in basketball game-related statistics among continental championships for U18 women, and (b) to identify game-related statistics that discriminate among the continents. The ANOVA yielded significant *F*-values for 13 of 16 variables and large effect size differences for 10 of 16 variables. The discriminant analysis yielded three significant discriminant functions. These results suggest that U18 women’s games are played differently in each continent.

Classification accuracy for the U18 women’s Asian championship was the highest among the four continental championships. Ball possessions, assists, and fouls greatly contributed to discriminating the U18 women’s Asian championship from the other continental championships. The number of ball possessions in the U18 women’s Asian championship was the highest among the four continental championships, whereas the numbers of assists and fouls were the lowest among the four continental championships. A high number of ball possessions indicates that the pace of the game was relatively fast, and a low number of assists indicates that most of the points were scored by individual plays. Interestingly, these performance profiles have similarly been observed in Asian championships for senior [3] and junior [5] men but not in senior women [4]. The previous study on senior women’s continental championships of 2017 showed that the number of assists in the Asian championship was the highest, and the number of ball possessions in the Asian championship was the second lowest among the four continental championships [4]. An important factor to consider when comparing the 2016 U18 and the 2017 senior women’s Asian championships is the difference in participating countries between the two championships. Two Oceanian countries, Australia and New Zealand, participated in the 2017 senior women’s Asian championship but not in the 2016 U18 women’s Asian championship because the FIBA has merged Oceanian and Asian championships since 2017. The participation of these two countries, especially the 4th-ranked Australia [27], might affect performance profiles of Asian championships. However, excluding the two countries from the analysis did not cause much differences in mean values of ball possessions (75.1 vs. 76.1) and assists (25.3 vs. 25.0) in the 2017 senior women’s Asian championship. Therefore, the difference between the U18 and the senior women’s Asian championships cannot be explained by the difference in participating countries. It should be noted that not only differences but also similarities were found between the U18 and the senior women’s Asian championships. Both the U18 and the senior women’s Asian championships showed the lowest numbers of fouls, free throw attempts, and turnovers among four continental championships. These results suggest that women’s games in Asia are offense-oriented and/or less physical.

Classification accuracy for the U18 women’s European championship was the second highest among the four continental championships. Point difference and ball possessions distinguished the U18 women’s European championship from the other continental championships. Large effect size differences were found in all pairwise comparisons between the U18 women’s European championship and the other continental championships for point difference (vs. Africa, *d* = 1.02; vs. America, *d* = 1.00; vs. Asia, *d* = 0.91) and ball possessions (vs. Africa, *d* = 1.08; vs. America, *d* = 1.46; vs. Asia, *d* = 2.21). The mean point difference and the number of ball possessions in the U18 women’s European championship were the lowest among the four continental championships. These results indicate that the U18 women’s European championship was characterized by a relatively slow pace and closely contested games. These characteristics have also been observed in European championships for men [3], women [4], and junior men [5]. Club-based player development systems in Europe [28] may have contributed to the formation of the style of European basketball, independent of age and sex.

Three-point field goals and free throws played a major role in discriminating the U18 women’s African championship from the other continental championships. The number of successful 3-point field goals in the U18 women’s African championship was the lowest among the four continental championships, and the number of unsuccessful free throws in the U18 women’s African championship was the highest among the four continental championships. In addition, when the numbers were converted to percentage values, both 3-point field goal and free throw percentages were the lowest among the four continental championships. These performance profiles have similarly been observed in African championships for men [3], women [4], and junior men [5]. Field goals are determined not only by players’ shooting skills but also by defensive pressure [29,30]. On the other hand, although psychological factors on free throws [20] cannot be ignored, shooting skills would be a major determinant of free throws, because players can shoot free throws without defensive pressure. Since free throw percentages in African championships are low, it can be said that African players have room for improvement in shooting skills.

The numbers of points scored, successful 3-point field goals, successful free throws, and assists in the U18 women’s American championship were the highest among the four continental championships. However, the number of unsuccessful 2-point field goals in the U18 women’s American championship was the lowest among the four continental championships. When the shooting-related statistics were converted to percentage values, the U18 women’s American championship ranked first in both 2- and 3-point percentages and ranked second in free throw percentage among the four continental championships. These results suggest that, on average, the American players have good shooting skills. In addition, good decision-making and passing skills, indicated by a high number of assists, may have contributed to the high shooting efficiency in the U18 women’s American championship. However, it should be noted that classification accuracy for the U18 women’s American championship was markedly low (47.5%). This result was in line with previous observations in American championships for senior women (43.8%) [4] and U18 men (45.0%) [5]. Low homogeneity in performance profiles would be one of the characteristics of American championships.

This study used the same method as in previous studies on continental championships for men [3], women [4], and U18 men [5]. Therefore, this study inevitably has the same limitations as the previous studies: we could not analyze detailed elements of the game, such as offensive [29,31] and defensive [32,33] strategies, shot types [34,35], and scoring dynamics [11,36], because the data were obtained only from box scores. However, using the same method has the advantage that we could easily compare the results among age and sex categories.

## 5. Conclusions

This study found large effect size differences in 10 of 16 game-related statistics and identified game-related statistics that discriminate among continental championships for U18 women. The Asian championship was discriminated from the other continental championships by ball possessions, defensive rebounds, assists, and fouls. The African championship was discriminated from the European championship by ball possessions, successful 3-point field goals, unsuccessful free throws, and turnovers, and from the American championship by ball possessions, unsuccessful 2-point field goals, successful 3-point field goals, successful free throws, and assists.

From a practical point of view, coaches of U18 women’s national teams can use this study to gain information on the opposing team based on the continent where the opposing team belongs. In addition, the results of this study would be valuable as part of scientific knowledge in the field of long-term development of female basketball players because studies on junior women are limited.

## Figures and Tables

**Table 1:** Participating teams and the number of games in the U18 women’s continental championships of 2016.

Continents	Teams	Games	Cases
Africa	8	24	48
	(Algeria, Angola, Egypt, Madagascar, Mali, Mozambique, Tunisia, Uganda)		
America	8	20	40
	(Brazil, Canada, Chile, Guatemala, Mexico, Puerto Rico, USA, Venezuela)		
Asia	12	36	72
	(China, Chinese Taipei, Hong Kong, India, Indonesia, Japan, Kazakhstan, Korea, Malaysia, Singapore, Sri Lanka, Thailand)		
Europe	16	56	112
	(Belgium, Croatia, Czech Republic, France, Hungary, Latvia, Lithuania, Netherlands, Russia, Serbia, Slovak Republic, Slovenia, Spain, Turkey)		
Total	44	136	272

**Table 2:** Means and SDs of game-related statistics with results of ANOVA and post hoc comparisons.

Statistics	AF		AM		AS		EU		ANOVA		AF-AM		AF-AS		AF-EU		AM-AS		AM-EU		AS-EU	
	Mean	SD	Mean	SD	Mean	SD	Mean	SD	*F*	*p*	*p*	*d*	*p*	*d*	*p*	*d*	*p*	*d*	*p*	*d*	*p*	*d*
PTS	52.6	22.8	68.2	21.6	60.4	18.6	61.1	12.3	5.80	**<0.01**	**<0.01**	0.70	0.11	0.38	**0.03**	0.53	0.15	0.40	0.18	0.46	1.00	0.05
PD	31.6	24.9	30.3	23.9	27.3	17.0	14.3	12.0	15.68	**<0.01**	1.00	0.05	1.00	0.21	**<0.01**	**1.02**	1.00	0.15	**<0.01**	**1.00**	**<0.01**	**0.91**
TBP	81.0	7.8	81.6	4.9	86.5	6.3	74.8	4.6	62.92	**<0.01**	1.00	0.10	**<0.01**	**0.81**	**<0.01**	**1.08**	**<0.01**	**0.84**	**<0.01**	**1.46**	**<0.01**	**2.21**
S2P	20.6	10.3	23.2	10.0	22.6	9.0	23.7	6.6	1.60	0.19	0.87	0.26	1.00	0.21	0.19	0.40	1.00	0.07	1.00	0.07	1.00	0.15
U2P	37.5	8.8	33.0	9.8	42.2	9.1	35.8	8.8	11.14	**<0.01**	0.12	0.49	**0.03**	0.52	1.00	0.19	**<0.01**	**0.99**	0.53	0.31	**<0.01**	0.72
S3P	3.7	2.6	7.1	3.7	4.9	2.7	6.8	3.5	15.05	**<0.01**	**<0.01**	**1.08**	0.34	0.42	**<0.01**	**0.96**	**<0.01**	0.73	1.00	0.08	**<0.01**	0.61
U3P	18.6	7.9	17.5	6.2	17.5	7.1	18.0	6.4	0.30	0.83	1.00	0.14	1.00	0.15	1.00	0.08	1.00	0.01	1.00	0.08	1.00	0.08
SFT	12.6	7.8	15.8	7.6	10.1	4.8	13.8	6.6	7.68	**<0.01**	0.14	0.41	0.26	0.40	1.00	0.17	**<0.01**	**0.96**	0.59	0.29	**<0.01**	0.62
UFT	13.4	6.8	9.2	4.9	6.9	3.6	7.3	4.3	22.31	**<0.01**	**<0.01**	0.72	**<0.01**	**1.27**	**<0.01**	**1.18**	0.11	0.54	0.23	0.41	1.00	0.10
DRB	39.1	7.7	34.8	9.6	31.1	8.2	38.7	7.1	16.29	**<0.01**	0.07	0.49	**<0.01**	**0.99**	1.00	0.05	0.11	0.42	0.05	0.50	**<0.01**	**1.01**
ORB	22.1	9.8	19.2	8.6	17.7	7.1	16.9	6.4	5.49	**<0.01**	0.47	0.31	**0.01**	0.53	**<0.01**	0.68	1.00	0.20	0.64	0.32	1.00	0.11
AST	15.2	7.5	19.9	8.4	10.5	5.9	18.2	6.0	25.20	**<0.01**	**<0.01**	0.60	**<0.01**	0.71	0.06	0.46	**<0.01**	**1.37**	0.92	0.26	**<0.01**	**1.29**
STL	16.3	9.5	16.1	5.6	13.3	7.3	13.0	4.9	4.28	**<0.01**	1.00	0.02	0.10	0.36	**0.03**	0.49	0.19	0.42	0.07	0.61	1.00	0.04
TO	31.3	11.9	28.3	7.5	23.7	9.1	24.0	6.1	11.14	**<0.01**	0.60	0.29	**<0.01**	0.73	**<0.01**	**0.88**	**0.04**	0.54	0.04	0.66	1.00	0.04
BLK	4.2	3.3	4.7	3.9	3.8	2.6	3.7	3.4	1.06	0.37	1.00	0.15	1.00	0.14	1.00	0.14	0.86	0.30	0.61	0.28	1.00	0.01
FC	22.5	6.9	21.1	6.1	16.8	4.4	22.8	5.5	18.76	**<0.01**	1.00	0.22	**<0.01**	**1.03**	1.00	0.05	**<0.01**	**0.84**	0.51	0.32	**<0.01**	**1.19**

PTS, points scored; PD, point difference; TBP, team ball possessions; S2P, successful 2-point field goals; U2P, unsuccessful 2-point field goals; S3P, successful 3-point field goals; U3P, unsuccessful 3-point field goals; SFT, successful free throws; UFT, unsuccessful free throws; DRB, defensive rebounds; ORB, offensive rebounds; AST, assists; STL, steals; TO, turnovers; BLK, blocks; FC, fouls committed. *p* ≤ 0.05 and *d* > 0.79 are presented in bold.

**Table 3:** Classification results of discriminant analysis.

Calculation	Continent	Predicted				Total
		AF	AM	AS	EU	
Count	AF	**31**	2	4	11	48
	AM	2	**19**	6	13	40
	AS	2	0	**68**	2	72
	EU	6	8	4	**94**	112
Percentage	AF	**64.6**	4.2	8.3	22.9	100
	AM	5.0	**47.5**	15.0	32.5	100
	AS	2.8	0.0	**94.4**	2.8	100
	EU	5.4	7.1	3.6	**83.9**	100

AF, Africa; AM, America; AS, Asia; EU, Europe. Accurate classifications are presented in bold.

**Table 4:** Discriminant functions with structural coefficients (SC) for each variable.

Statistics	Function 1	Function 2	Function 3
Eigenvalue	1.60	0.72	0.21
Wilks’ Lambda	0.18	0.48	0.82
Chi-square	442.6	192.5	50.7
*p*	<0.01	<0.01	<0.01
Proportion of trace (%)	63.2	28.4	8.4
Canonical correlation	0.78	0.65	0.42
Team ball possessions	**−0.59**	**−0.42**	**0.39**
Successful 2-point field goals	0.02	0.15	0.07
Unsuccessful 2-point field goals	−0.24	−0.03	**−0.36**
Successful 3-point field goals	0.15	**0.37**	**0.40**
Unsuccessful 3-point field goals	0.03	−0.03	−0.07
Successful free throws	0.19	0.04	**0.34**
Unsuccessful free throws	0.14	**−** **0.55**	−0.06
Defensive rebounds	**0.32**	−0.03	−0.29
Offensive rebounds	0.03	−0.29	0.03
Assists	**0.38**	0.12	**0.44**
Steals	0.04	−0.22	0.21
Turnovers	0.10	**−0.38**	0.15
Blocks	0.02	−0.07	0.19
Fouls committed	**0.36**	−0.01	−0.11

|SC| ≥ 0.30 in bold.
